# Overexpression of Cu/Zn Superoxide Dismutase (Cu/Zn SOD) in *Synechococcus* *elongatus* PCC 7942 for Enhanced Azo Dye Removal through Hydrogen Peroxide Accumulation

**DOI:** 10.3390/biology10121313

**Published:** 2021-12-10

**Authors:** ShylajaNaciyar Mohandass, Mangalalakshmi Ragavan, Dineshbabu Gnanasekaran, Uma Lakshmanan, Prabaharan Dharmar, Sushanta Kumar Saha

**Affiliations:** 1 Department of Marine Biotechnology, National Facility for Marine Cyanobacteria (Sponsored by DBT, Govt. of India), School of Marine Sciences, Bharathidasan University, Tiruchirappalli 620024, Tamil Nadu, India; shylajanachiyar@gmail.com (S.M.); mangalalakshmi.r@vit.ac.in (M.R.); dineshbty1@gmail.com (D.G.); lumaprabakar@yahoo.com (U.L.); nfmc@bdu.ac.in (P.D.); 2 Shannon Applied Biotechnology Centre, Technological University of Shannon, Moylish Park, V94 E8YF Limerick, Ireland

**Keywords:** superoxide dismutase (SOD), cyanobacteria, copper/zinc superoxide dismutase (Cu/Zn SOD), hydrogen peroxide (H_2_O_2_), *Synechococcus elongatus* PCC 7942

## Abstract

**Simple Summary:**

Synthetic azo dyes are used in various industries like apparel, food, paper, etc. The improper discharge of these recalcitrant dyes poses a serious threat to the environment. However, in nature, certain microorganisms including cyanobacteria can degrade these recalcitrant compounds by producing various reactive oxygen species (ROS), particularly by hydrogen peroxide (H_2_O_2_). This study aimed at developing a genetically modified cyanobacterium for better H_2_O_2_ accumulation that results due to superoxide radicals dismutation by enzyme superoxide dismutase (SOD). The modified strain (MS-*sodC*^+^) was created by integrating Cu/Zn SOD gene (*sodC*) from another cyanobacterium, and its expression was driven by a strong constitutive *psbA* gene promoter. The modified strain (MS-*sodC*^+^) resulted in over-accumulation of H_2_O_2_ during azo dye treatment with a higher rate of dye removal than the wild-type strain (WS-*sodC*). Therefore, to encourage cyanobacteria for biodegradation of toxic azo dyes faster than the natural rate enhanced H_2_O_2_ accumulation through SODs, overexpression may serve as a valuable bioremediation tool.

**Abstract:**

Discharge of recalcitrant azo dyes to the environment poses a serious threat to environmental health. However certain microorganisms in nature have developed their survival strategies by degrading these toxic dyes. Cyanobacteria are one such prokaryotic, photosynthetic group of microorganisms that degrade various xenobiotic compounds, due to their capability to produce various reactive oxygen species (ROS), and particularly the hydrogen peroxide (H_2_O_2_) when released in their milieu. The accumulation of H_2_O_2_ is the result of the dismutation of superoxide radicals by the enzyme superoxide dismutase (SOD). In this study, we have genetically modified the cyanobacterium *Synechococcus* *elongatus* PCC 7942 by integrating Cu/Zn SOD gene (*sodC*) from *Synechococcus* sp. PCC 9311 to its neutral site through homologous recombination. The overexpression of *sodC* in the derivative strain was driven using a strong constitutive promoter of the *psbA* gene. The derivative strain resulted in constitutive production of *sodC*, which was induced further during dye-treated growth. The genetically engineered *Synechococcus* *elongatus* PCC 7942 (MS-*sodC*^+^) over-accumulated H_2_O_2_ during azo dye treatment with a higher dye removal rate than the wild-type strain (WS-*sodC^−^*). Therefore, enhanced H_2_O_2_ accumulation through SODs overexpression in cyanobacteria may serve as a valuable bioremediation tool.

## 1. Introduction

Synthetic dyes including azo dyes are recalcitrant compounds and their usage in various industries like apparel, food, paper, etc., have increased significantly, which pose a serious threat to the environment. These dyes are chemically and photolytically stable, hence are highly persistent in natural environments, and their presence in water and soil is undesirable due to their appearance and toxicity [1]. These dyes resist various conventional degradation methods that suffer from numerous disadvantages like operational costs, incomplete removal, residue accumulation, and need for high energy. Hence, the usage of microbial communities is considered economically feasible and environmentally friendly [2]. Many microbes like bacteria, fungi, and algae were reported for their ability to neutralize and remove these dyes effectively [3].

Cyanobacteria, the unique photosynthetic prokaryotes believed to be responsible for oxygenating the earth, have a lot of credit over other microorganisms in a view of their trophic independence for nitrogen and carbon, and their ability to utilize the nitrogen, phosphorus, carbon, and sulfur sources from the degraded products [2,4]. Cyanobacteria, being oxygenic photosynthetic organisms, inevitably generate various reactive oxygen species (ROS) by their photosynthetic electron transport [5]. These ROS include free radicals such as superoxide anion (O^2•−^), hydroxyl radical (^•^OH), as well as non-radical molecules such as hydrogen peroxide (H_2_O_2_) and singlet oxygen (^1^O_2_, delta state), and their induced production are an unavoidable consequence of stress conditions [6]. Production of H_2_O_2_ by cyanobacteria is not surprising, as cyanobacteria photo produce O_2_ at a higher rate. The photoreduction of two molecules of O_2_ requires two superoxide radicals to form one molecule of H_2_O_2_ catalyzed by the enzyme superoxide dismutase (SOD) [7].

SODs in cyanobacteria can be classified into three types based on cofactors such as Fe, Mn, or Cu and Zn, present at the active sites [8]. In comparison with the canonical isoforms of SODs, it was observed that Cu/Zn SOD is structurally distinct from Fe and Mn SOD. Cu/Zn SOD is considered to be the most important of the three SODs because of its role in preventing ROS-generated cell damage, and the death in aerobically growing organisms [9]. Interestingly, cyanobacterium *Anacystis nidulans* 6301 showed the ability to mitigate photooxidative damage when human Cu/Zn SOD was overexpressed under the control of the native *rbc* promoter on a shuttle vector. Under optimum growth conditions, an approximate 18-fold-increased SOD enzyme activity with a Cu/Zn SOD protein level of about 3% of the total soluble protein was recorded [10]. Studies reported that an increase or change in SOD activity will impact H_2_O_2_ accumulation leading to a concentration gradient, and the H_2_O_2_ flux that results in subsequent activation of particular redox-sensitive pathways [11]. Since H_2_O_2_ is uncharged, more stable, and can traverse through the membranes freely, it is a more versatile signalling molecule [12].

Cyanobacteria maintain their intracellular antioxidant levels by excreting H_2_O_2_ into the environment. Light-dependent excretion of H_2_O_2_ has been well documented in various strains of cyanobacteria [5,13,14]. If none of the H_2_O_2_ is decomposed within the cells, then one would expect sustained excretion of about 50 μmol H_2_O_2_ mg^−1^ chlorophyll based upon the observed rates of O_2_ photoreduction [15]. The beneficial role of ROS, especially H_2_O_2_, in water treatment, cleaning, decontamination, and remediation applications are well established [16]. However, no attempt has so far been made to channel and exploit the ROS, particularly H_2_O_2_, produced by cyanobacteria for dye removal.

Hence, an attempt has been made in this study to genetically modify the model cyanobacterium *S. elongatus* PCC 7942 with Cu/Zn SOD gene integration, ultimately for stable overproduction of H_2_O_2_ and to use this derivative strain for removal of azo dye.

## 2. Materials and Methods

### 2.1. Cyanobacterium and E. coli Growth Conditions

Cyanobacterium *Synechococcus elongatus* PCC 7942 used in this study was obtained as one of the components of GeneArt^®^ *Synechococcus* engineering kit (Invitrogen, Carlsbad, CA, USA). The frozen cells removed from −80 °C were placed on ice to avoid temperature fluctuations followed by gentle freeze–thawing at 34 °C. The thawed *S. elongatus* PCC 7942 cells were then plated on 1% agar containing BG-11 medium [17] and incubated for a week under continuous low light (20 μmol photons m^2^ s^−1^) at 27 ± 2 °C. Then, the green lawn was inoculated to 10 mL BG-11 liquid medium in glass tubes, and incubated for another week under continuous medium light (40 μmol photons m^2^ s^−1^) at 27 ± 2 °C. Finally, the wild-type *S. elongatus* PCC 7942 and its derivative strains were grown in 250 mL Erlenmeyer flasks containing 100 mL of BG-11 medium for their active growth at 27 ± 2 °C under continuous illumination of 70 μmol photons m^−2^ s^−1^.

*E. coli* TOP10 and its derivative strains were grown at 37 °C with LB medium in 1–5 mL tubes and agar plates with 100 rpm in shaking at controlled condition, supplemented with appropriate antibiotics [18]. The cell density with OD_600_ nm greater than 1 was subjected to experiments.

### 2.2. Vector and Primers Used for Cloning of sodC

The GeneArt^®^ *Synechococcus* TOPO^®^ Engineering Kit was obtained from Invitrogen, Thermo Fisher Scientific, Carlsbad, CA, USA, which contains the pSyn_6 vector (4461 bp) designed to facilitate rapid cloning and expression of the gene of interest (GOI) in *Synechococcus elongatus* PCC 7942. The pSyn_6 vector has NS1 (neutral site 1) homologous recombination sites, a strong constitutive promoter of *psbA* gene (encoding photosystem II protein D1) to drive the high-level expression of *sodC*, antibiotic spectinomycin resistance gene for selection in *E. coli* and the derivative *Synechococcus elongatus* PCC 7942 strains, pUC origin for the maintenance of cloned pSyn_6 plasmid in *E. coli*, and RP4/RK2 *bom* site for conjugation (Invitrogen^TM^ by Life Technologies^TM^, Carlsbad, CA, USA).

Cu/Zn SOD gene (*sodC*) from *Synechococcus* sp. PCC 9311 (NCBI accession number: CP000435.1, sequence length 528 bp) (Supplementary Figure S1) was in vitro synthesized with engineered restriction sites BglII and NdeI, (Genscript, Hyderabad, India). Primer set *sodC*_F (5′-GCACGATTGAGGTCACGATT-3′) and *sodC*_R (5′-CGCTTGTTGTGGTGTTGC-3′) were designed and synthesized (Eurofins, Bangalore, India) to confirm the transformation, which anneals and amplify the *sodC* flanked by NS1a and NS1b sites.

### 2.3. E. coli Transformation and PCR Confirmation

Synthesized *sodC* gene and pSyn_6 vector were digested with BglII and NdeI individually in two steps, the digested DNA fragments were resolved through 1.2% agarose gel electrophoresis, and extracted by gel elution kit (QIAquick^®^ Gel Extraction Kit, New Delhi, India). The purified restriction digested DNA fragments (*sodC* gene) were ligated with pSyn_6 vector (3:1 ratio) by T4 DNA ligase following the recommended protocol (New England Biolabs, Chennai, India). Then, one-shot chemical transformation protocol was followed as described in the kit to transform the ligated DNA mixture to one-shot chemically competent *E. coli* TOP10 cells. These cells were then plated on LB agar medium containing 100 µg ml^−1^ spectinomycin and incubated overnight at 37 °C for the selection of spectinomycin-resistant transformed colonies. A total of six random colonies were picked and inoculated individually in 2 mL of LB broth medium with 100 µg ml^−1^ spectinomycin, and incubated overnight at 37 °C with shaking at 100 rpm. Then, the biomass pellets were extracted using Purelink HQ mini plasmid DNA purification kit (Invitrogen, Carlsbad, CA, USA). The plasmid DNA was quantified spectrophotometrically and PCR amplified to confirm the transformed clone containing *sodC* gene. PCR amplification was carried out using primers *sodC*_F and *sodC*_R for 30 cycles of the following three-steps (30 s at 94 °C, 30 s at 57 °C, and 1 min at 74 °C) and a final step of 10 min at 74 °C (Eppendorf^®^ Mastercycler^®^ Pro Thermal Cyclers, Chennai, India).

### 2.4. Transformation of sodC to S. elongatus PCC 7942 by Homologous Recombination

Firstly, 1.5 mL of actively growing *S. elongatus* PCC 7942 cells with OD_750_ greater than 1 was collected and washed twice with BG-11 medium by centrifugation at 14,000 rpm for 1 min. Then, the cell pellets were resuspended in 100 µL of BG-11 medium and added 100 ng of plasmid DNA (pSyn_6 vector + *sodC*), where 100 ng of pSyn_6 vector alone served as a negative control. *S. elongatus* is a naturally transformable cyanobacterium with high efficiency at the log phase of growth (OD_750nm_ of 1–2). The transformation mixture was incubated at 34 °C in dark for 4 h followed by plating of the transformation mixture (20 and 80 µL) on BG-11 containing 1.5% agar and 10 µg mL^−1^ spectinomycin. Plates were placed under continuous illumination of 70 μmol photons m^−2^ s^−1^ at room temperature (25–30 °C) for 7 days. Colonies appeared on the 5th day and the positive clones were tested (six individual colonies) by colony PCR using the primer set and PCR amplification conditions mentioned above.

### 2.5. Experimental Conditions and Growth Analysis

*S. elongatus* PCC 7942 transformed with *sodC* gene was considered as modified strain (MS-*sodC*^+^) and non-transformed as the wild-type strain (WS-*sodC^−^*) throughout the manuscript. For experiments, MS-*sodC*^+^ (three individual positive colonies) and WS-*sodC^−^* were grown in BG-11 medium at 27 ± 2 °C at 20 μmol photons m^2^ s^−1^ light intensity. The growth medium of MS-*sodC*^+^ contained 10 μg mL^−1^ spectinomycin. Mid-log cultures were centrifuged at 14,000 rpm for 5 min, the cell pellets were washed with BG-11, and resuspended in the same medium. These resuspended cells were used as inoculum for all the experiments in the BG-11 medium. The growth was analyzed on the 7th day. *E. coli* cells were cultured at 37 °C in Luria–Bertani (LB) broth or on LB agar plates containing appropriate antibiotics**.** The diazo dye, Acid Black 1 (C.I. 20470, Sigma Aldrich, Chennai, India) was used in the study. A stock solution was prepared by dissolving the dye in deionized water and sterilizing it by membrane filtration (0.2 μM, Millipore, Chennai, India) before use.

### 2.6. Preparation of Enzyme Extracts and Enzyme Activity

SOD activity of MS-*sodC*^+^ and WS-*sodC^−^* *S. elongatus* PCC 7942 cells was examined at the 12th and 24th hours of incubation under dye treated and untreated conditions. Each cyanobacterial pellet was extracted with ice-cold 25 mM Tris-Cl, pH 7.0 buffer using a prechilled pestle and mortar at 4 °C [19]. Crude extracts were clarified two times by centrifugation at 15,000 rpm for 15 min at 4 °C. The clear supernatants obtained served as enzyme sources. The protein concentration in the extract was estimated following the method described earlier [20] and 250 μg of total proteins was loaded in each well along with sample buffer for native-PAGE. Then electrophoresis was carried out at 18 ± 1 °C with polyacrylamide gels (size 1.5 mm × 14 cm × 14 cm) in Tris-glycine buffer (pH 8.3) under non-denaturing and non-reducing conditions [21]. The samples were electrophoresed at 100V through the stacking gel (6%) and at 120V through the separating gel (10%) until the bromophenol blue dye reached the bottom of the gel.

Activity staining for the identification of Cu/Zn SOD was carried out using the method described earlier [22]. To find the Cu/Zn SOD enzyme activity two gels containing identical samples were incubated by soaking: (1) in 50 mL of 50 mM, Tris HCl (pH 8) buffer containing 2 mM KCN (inhibitor for Cu/Zn SOD), and (2) in 50 mL of 50 mM Tris HCl (pH 8) buffer without any inhibitors (for total SOD profile) for 20 min in the dark. Then, both gels were rinsed gently with the same Tris buffer before soaking the gels to staining solution. For the identification of the SOD banding profile, the gels were soaked in a staining solution containing 50 mL of 50 mM Tris HCl (pH 8), 10 mg NBT (Sigma, St. Louis, USA), 1 mg EDTA (Sigma, St. Louis, USA), and 2 mg riboflavin (Sigma, St. Louis, USA) for 30 min in the dark at room temperature (25 °C). After dark incubation, the gel was illuminated on a lightbox with white fluorescent light (80 µmol photons m^−2^ s^−1^) until achromatic bands appeared [23].

### 2.7. Decolorization and Degradation Assay

The culture supernatants of dye-treated and untreated MS-*sodC*^+^ and WS-*sodC^−^* *S. elongatus* PCC 7942 were harvested by centrifugation at 15,000 rpm for 8 min after the 12th and 24th hours of cultivation. The clear supernatants were used as the sample for decolorization and degradation assay. Then, 5 mL of the clear supernatants were used to read the absorption peak at 618 nm in a spectrophotometer (Varian cary-100, New Life Scientific Inc., Chennai, India) and the percentage of decolorization was calculated as follows:

Decolorization (%) = [(B − A)/A] × 100

where A = initial absorbance at zero hour, B = final absorbance.

For HPLC analysis, 20 µL of clear supernatants were filtered using a 0.22 μM filter and used for injection. The azo dye sample was eluted using Zorbax C18 extended column (250 mm × 4.6 mm) using methanol as the mobile phase with a flow rate of 1 mL min^−1^. The eluent product was monitored at regular intervals by their absorbance at 557 nm with a UV detector, and the extend of dye degradation was calculated using the formula:

% degradation = (peak area of untreated sample − peak area of treated sample)/peak area of untreated sample.

### 2.8. Determination of H_2_O_2_ Production

H_2_O_2_ concentrations were measured in the growth medium of dye-treated and untreated MS-*sodC*^+^ and WS-*sodC^−^ S.* *elongatus* PCC 7942 cultures. Production of H_2_O_2_ was determined from the chromogen formed by 4 aminoantipyrine and phenol [24]. The change in absorbance was measured at 505 nm and expressed as µmol H_2_O_2_ µg Chl *a*^−1^ h^−1^. The values represented are the average of triplicates.

### 2.9. Statistical Analysis

A minimum of six transformant colonies (*E. coli* and *S.* *elongatus* PCC 7942) were verified by PCR amplification as described above. All experiments related to dye decolorization and degradation were carried out with three individuals cyanobacterial transformants, and the results presented are expressed as averages ± standard error (SE) of triplicate samples. The native-PAGE for the expression of Cu/Zn SOD was analyzed qualitatively comparing their achromatic banding profiles.

## 3. Results and Discussion

Improper discharge of synthetic azo dyes poses a serious threat to the environment because of its recalcitrant nature due to the presence of highly reactive amine (N=N) and sulfonic (–SO_3_^−^) groups [25,26,27]. Therefore, an emerging concern is to come up with effective methods to remove the dyes before releasing them into the environment [28]. Microbes-mediated azo dye remediation through powerful reactive oxygen species (ROS), such as superoxide radical anion (O_2_^−^), hydroxyl radical (OH), and hydrogen peroxide (H_2_O_2_), is considered more effective because of its eco-friendly nature [29]. These ROS are produced naturally in all photoautotrophic organisms including cyanobacteria [30], which are oxygen-evolving photosynthetic prokaryotes. Cyanobacteria serve as potent ROS-producing candidates and were well documented for their wide adaptability to mineralize the synthetic dye [2,31].

H_2_O_2_ is a powerful oxidant, which is produced as a by-product during the breakdown of superoxide by SODs, the first enzyme in the line of defense against oxidative stress [29]. Continuous production of H_2_O_2_ will serve as an attractive means to bio-converse the solar radiation into storable chemical energy a special condition of its involvement in eliminating the toxicity [32]. It was well documented that cyanobacteria photoreduces O_2_ with the concomitant formation of H_2_O_2_ through the typical Mehler reaction [33,34]. However, certain cyanobacteria were found to produce H_2_O_2_ during light-independent reactions [35]. The efficiency of photosynthetic production of H_2_O_2_ by Mehler reaction in cyanobacteria depends on the metabolic conditions of cells due to culture age [34]. The constitutive overexpression of the SOD might help accumulate H_2_O_2_ in the organism’s vicinity that can be utilized as a biologically produced oxidizing agent to remediate the dye contamination. However, it also should be considered that H_2_O_2_ photoproduction in cyanobacteria cannot be sustained continually because of inhibition to photosynthesis due to high oxygen concentrations or increased concentration of H_2_O_2_ [34,36]. Therefore, H_2_O_2_ produced may need to be removed continuously from the vicinity of the cells to support continuous production as well as their effective use as an oxidative agent. This approach might provide a reliable, economical, and environmentally friendly solution for azo dye removal. Therefore, an attempt was made to genetically modify the model cyanobacterium *S.* *elongatus* PCC 7942 to overproduce the enzyme Cu/Zn SOD, one of the versatile SODs for continuous release of H_2_O_2_ into medium, and thus to develop a biological solution for azo dye remediation.

### 3.1. Transformation and Expression of Cu/Zn SOD in S. elongatus PCC 7942

**Figure 1:**
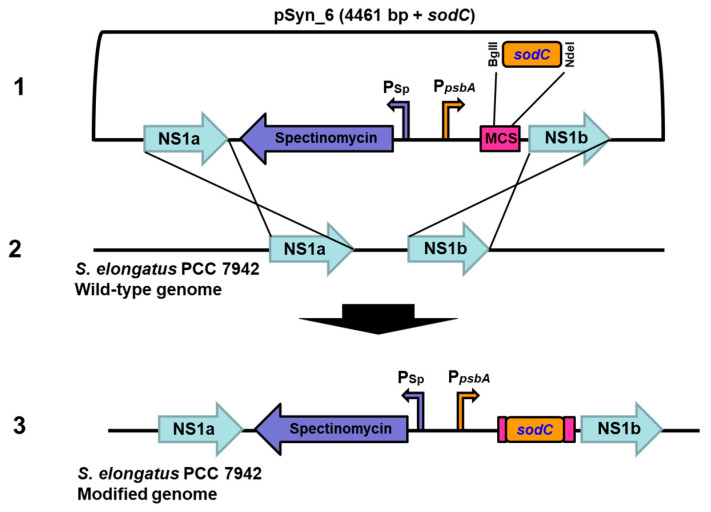
Vector and integration maps of the *sodC* gene in neutral sites of *S. elongatus* PCC 7942. Schematic showing pSyn_6 vector containing *sodC* flanked by the neutral sites (**1**) and its mode of homologous recombination with NS1a and NS1b of *S. elongatus* PCC 7942 (**2**), resulting in genetic integration of the *sodC* gene in *S. elongatus* PCC 7942 genome (**3**). P_sp_, spectinomycin promoter; P*_psbA_*, constitutive promoter of *psbA* gene; MCS, multiple cloning site.

The in vitro synthesized *sodC* gene from *Synechococcus* sp. PCC 9311 that codes for Cu/Zn SOD was ligated to pSyn_6 expression vector and the transformed *E. coli* Top 10 cells were selected on agar plates (Supplementary Figure S2). The cloned vector containing *sodC* was transformed to *S. elongatus* PCC 7942 and the transformed colonies on agar plate were selected with antibiotic spectinomycin (Supplementary Figure S3). The pSyn_6 vector containing the *sodC* has a sequence homologous to the neutral site of *S. elongatus* PCC 7942, and hence the resultant cyanobacterial transformants with spectinomycin resistance are due to a double homologous recombination event (Figure 1). The integration of *sodC* to the neutral site of *S. elongatus* PCC 7942 transformants was substantiated by the PCR amplification of *sodC* by employing specific primers (Supplementary Figure S4).

**Figure 2:**
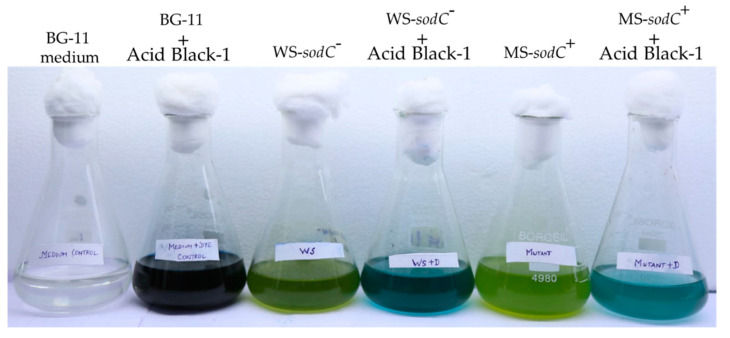
Dye-treatment experimental set-up showing representative control flasks (BG-11 medium alone, and BG-11 medium with Acid Black-1 dye), control strains (WS-*sodC*^−^ and MS-*sodC*^+^ in BG-11 medium), and strains (WS-*sodC*^−^ and MS-*sodC*^+^) grown for 24 h in the BG-11 medium containing Acid Black-1 dye.

**Figure 3:**
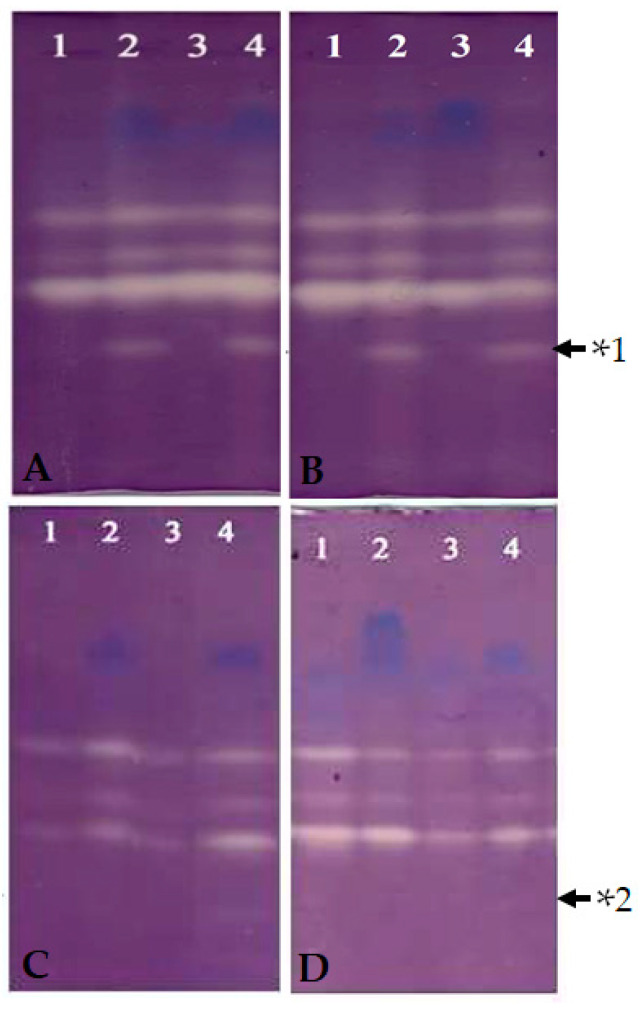
Expression-banding profile of superoxide dismutases in MS-*sodC*^+^ and WS-*sodC*^−^ *S.* *elongatus* PCC 7942. Crude protein was extracted at the 12th and 24th hours from azo dye treated MS-*sodC*^+^ and WS-*sodC*^−^ *S.* *elongatus* PCC 7942 cells. Samples loaded in each well possess 250 µg of total protein. Native–PAGE gel was activity stained simultaneously with (**C**,**D**) and without (**A**,**B**) inhibitor KCN to identify the expression of Cu/Zn SOD. Constitutive expression of Cu/Zn SOD was observed in the MS-*sodC*^+^ (Lane 2 of (**A**,**B**)), which was found to be significantly higher under dye treated growth (Lane 4 of (**A**,**B**)). However, no Cu/Zn SOD band was found in WS-*sodC*^−^ in the corresponding position confirms its absence of expression. Upper panel (12th hour (**A**) and 24th hour (**B**)): Activity staining of untreated Native gel. Lane 1—Without dye, WS-*sodC*^−^ Lane 2—Without dye, MS-*sodC*^+^; Lane 3—With dye, WS-*sodC^−^*; and Lane 4—With dye, MS-*sodC*^+^. Lower panel (12th hour (**C**), and 24th hour (**D**)): Activity staining of KCN treated Native gel. Lane 1—Without dye, WS-*sodC*^−^; Lane 2—Without dye, MS-*sodC*^+^; Lane 3—With dye, WS-*sodC^−^*; and Lane 4—With dye, MS-*sodC*^+^. *1, Cu/Zn SOD band appeared in MS-*sodC*^+^ strain and *2, same Cu/Zn SOD band disappeared due to inhibition of enzyme activity by KCN treatment of the Native gel.

The expression banding pattern of SODs of WS-*sodC^−^* and MS-*sodC*^+^ *S. elongatus* PCC 7942 strains in growth medium without dye and with dye (Figure 2) was analyzed at the 12th and 24th hour through native gel activity staining (Figure 3). The gels showed the expressions of three distinct SOD bands in WS-*sodC^−^* strain in growth medium without and with dye., There was an appearance of lower molecular weight band (Figure 3, upper panel) and disappearance of the same band in gel soaked with inhibitor KCN in MS-*sodC*^+^ strain in growth medium without and with dye. This inhibitor assay confirms the expression of the Cu/Zn SOD in MS-*sodC*^+^ strain (Figure 3, lower panel). The molecular weight of Cu/Zn SOD is approximately 16-23 kDa, whereas Fe- and Mn-SOD range between 22 and 34 kDa; hence, the band position of appeared SOD band in MS-*sodC*^+^ strain suggests a lower molecular weight Cu/Zn SOD [8]. The appearance of the band in activity native gel when MS-*sodC*^+^ strain was grown in medium without dye confirms that the introduced Cu/Zn SOD gene was expressed in active form constitutively. The expression of this Cu/Zn SOD was slightly increased after 24 h of growth in dye-containing medium (Figure 3B). A similar report [37] documented the increased expression of SOD by twofold in cyanobacterium *Leptolyngbya valderiana* BDU 20,041 when grown in medium containing acid black dye to alleviate the oxidative stress and for sustenance in growth. However, to make a very efficient H_2_O_2_ producing system, it might be necessary to enhance the expression of target SODs as that of heterologous expression of human Cu/Zn SOD in *Anacystis nidulans* 6301 under the control of strong light-driven Ribulose-1,5-bisphosphate Carboxylase/oxygenase (*rbc*) promoter [10].

### 3.2. Hydrogen Peroxide Accumulation in Transgenic S. elongatus PCC 7942

To evaluate the overproduced SOD’s participation in the degradation process, the crude protein extracts of WS-*sodC^−^* and MS-*sodC*^+^ strains grown without and with dye treatment were tested for H_2_O_2_ production in vitro. SODs are the enzymes that actively determine the concentration of O^2•−^ and H_2_O_2_ levels, and are therefore important in antioxidant defense mechanisms [38].

**Figure 4:**
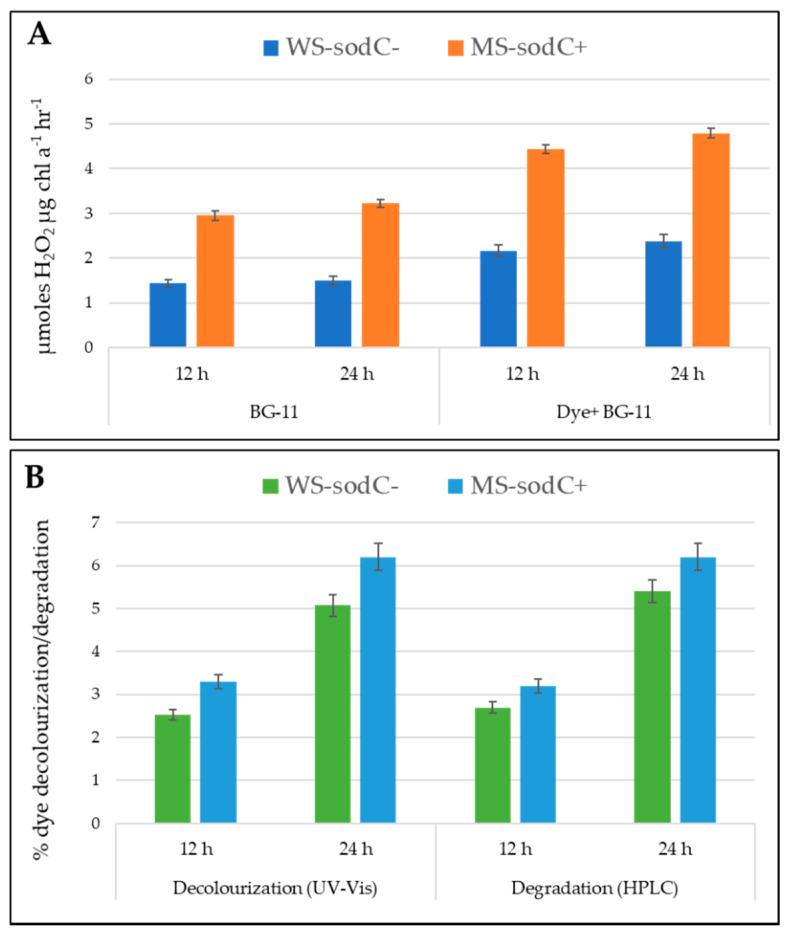
The figure depicts the H_2_O_2_ production (**A**), decolorization, and degradation (**B**) properties of WS-*sodC*^−^ and MS-*sodC*^+^ *S.* *elongatus* PCC 7942 strains after the 12th and 24th hours of cultivation with azo dye, Acid black-1. The MS-*sodC*^+^ showed higher H_2_O_2_ production, decolorization, and degradation when compared with WS-*sodC*^−^ strain.

Figure 4 shows the relative content of H_2_O_2_ accumulation in a time-dependent, dye-dependent, and strain-dependent manner. The crude enzyme obtained from WS-*sodC^−^* strain produced 1.437 and 1.502 µmol H_2_O_2_ μg chl *a*^−1^ h^−1^, respectively, at the 12th and 24th hours of incubation. It was reported earlier that cyanobacterium *Anacystis nidulans* can produce only 16–110 µmol H_2_O_2_ per mg chlorophyll per hour under different growth conditions [34]. Whereas in this study, the same strain when treated with dye showed a 0.51- and 0.58-fold-induced increase in the production of H_2_O_2_, respectively, at the 12th and 24th hour. Similar activity was observed in *Oscillatoria curviceps* BDU92191, which produced greater excess H_2_O_2_ than normal with sustained growth and tolerance under dye treated stress conditions [2]. Moreover, in plants, H_2_O_2_ was reported to produce and accumulate as an antioxidant defense under adverse environmental conditions [39,40]. In the present study, the genetically modified MS-*sodC*^+^ *S. elongatus* PCC 7942 strain showed about a onefold-increased production of H_2_O_2_ than the WS-*sodC^−^* strain, irrespective of incubation time and dye-treatment. This substantiated that the introduced Cu/Zn SOD enzyme was constitutively expressed in the modified strain with enhanced H_2_O_2_ production both in growth medium without and with dye. Even though Cu/Zn SOD in MS-*sodC*^+^ was constitutively expressed, dye stress further enhanced the accumulation of H_2_O_2_ more effectively than the WS-*sodC^−^* strain. The slightly induced expression of the Cu/Zn SOD band in dye treated cells of MS-*sodC*^+^ after 24 h incubation, possibly indicated oxidative stress created by the presence of dye in the growth medium (Figure 3B). Similarly, in *Elsholtzia haichowensis*, Cu stress resulted in increased activity of Cu/Zn SOD isoforms, while no significant change in Mn SOD activity was recorded. Overall, increased SOD activity enhanced the production and accumulation of high levels of H_2_O_2_ within the cell walls or the outer side of the plasma membrane during Cu stress conditions [9]. However, under Cd stress, *Arabidopsis* plants overexpressing Sa Cu/Zn SOD (Cu/Zn SOD gene from *Sedum alfredii*) showed the differences in the accumulation of O^2•−^ and H_2_O_2_ [41].

### 3.3. Role of H_2_O_2_ in Transgenic S. elongatus PCC 7942

In general, H_2_O_2_ is produced in response to oxidative stress and is positively used to activate the multiple responsive genes to cope with the environmental stress conditions [42]. *Mycorrhiza* was reported to induce more H_2_O_2_ effluxes of the plant roots, and thereby maintaining the H_2_O_2_ gradient and alleviating the oxidative damage of drought stress in the host plant [43]. H_2_O_2_ can act as a physiological regulator of metabolic activities and signaling pathways that depend on the redox state of their components, and the distance travel by H_2_O_2_ is inversely related to the targets it encounters in the pathway [44]. The WS-*sodC^−^* strain accumulated a maximum of 2.38 µmol H_2_O_2_ μg chl *a*^−1^ h^−1^ in dye-containing medium with a maximum of 5.07% and 5.4% of dye decolorization and dye degradation respectively at 24th h. While under similar growth conditions, the MS-*sodC*^+^ strain accumulated a maximum of 4.79 µmol H_2_O_2_ μg chl *a*^−1^ h^−1^ with a maximum of 6.2% of dye decolorization and dye degradation at 24th h (Figure 4). This suggests that the higher accumulation and production of H_2_O_2_ directly increases the decolorization and degradation rate of azo dye in MS-*sodC*^+^ *S. elongatus* PCC 7942. Tabaï et al. [45] reported the potential oxidation of azo dye by using H_2_O_2_ in the aqueous solution and its decolorization efficiency was dependent on the concentration of the H_2_O_2_ tested. In another study with dye-treated aqueous solution, the increased rate of dye decolorization through oxidation was found with increased H_2_O_2_ concentration [46]. Similarly, enhanced removal of C.I. Acid Orange 7 from aqueous solution was achieved by the addition H_2_O_2_ up to a concentration of 0.8 mM [47]. The MS-*sodC*^+^ strain transformed with *sodC* induced the H_2_O_2_ accumulation, which is more helpful in enhanced dye decolorization and degradation than the wild-type strain. However, the application of this approach of bioremediation needs further optimization including the cyanobacterial cellular density to achieve a higher concentration of H_2_O_2_ to be released and metal co-factors that determine the effectiveness of H_2_O_2_ in the wastewater system. Manganese porphyrins in water act as catalysts for azo dye decolorization by H_2_O_2_ under mild environmental conditions and the rate of dye decolorization was found to be associated with the structure of the manganese porphyrins [48,49]. Decomposition of various dyes through accumulated H_2_O_2_ from cyanobacteria has a lot of positive environmental benefits and commercial values, which wait for their bioremediation applications.

## 4. Conclusions

The present study reveals the potential of transgenic *S. elongatus* PCC 7942 to constitutively overexpress the *sodC* gene for enhanced H_2_O_2_ accumulation and, thus, help in azo dye biodegradation. The same genetic modification technique could be extended for the treatment of different complex effluents; however, it might require optimization of appropriate gene expressions under the control of a much stronger promoter for the abatement of pollution.

## Data Availability

The data obtained in this study are available within the article as well as supplementary information. However, any other standard technical information, which if not available unintentionally can be obtained upon request from the corresponding authors.

## Supplementary Materials

The following are available online at https://www.mdpi.com/article/10.3390/biology10121313/s1, Figure S1: Sequence of gene of interest *sodC* obtained from the *S. elongatus* PCC 9311 used in this study, Figure S2: Colonies in plate shows the *sodC* transformation in *E. coli* TOP10 in medium containing antibiotic spectinomycin, Figure S3: Colonies in plate shows the *sodC* transformation in *S. elongatus* PCC 7942 in medium containing antibiotic spectinomycin, Figure S4: PCR confirmation of *sodC* gene integration at neutral sites of *S. elongatus* PCC 7942.
