# Supplementary material for: Overexpression of Cu/Zn Superoxide Dismutase (Cu/Zn SOD) in Synechococcus elongatus PCC 7942 for Enhanced Azo Dye Removal through Hydrogen Peroxide Accumulation
Source: Biology (Basel). 2021 Dec 10;10(12):1313. doi: 10.3390/biology10121313 (PMC8698522; doi:10.3390/biology10121313)
Supplement: Supplementary file 1 [file biology-10-01313-s001.zip › biology-1497275-supplementary.pdf]

Supplementary Figures:

>WP_011619688.1 superoxide dismutase family protein [*Synechococcus* sp. CC9311]
MYRLGALLALCLALLMPATVQASTIEVTINSINTEGIGESIGTISARDTDQGLVIIPELSGLSEGEHGFH
LHAGDQCAPQTNSEGASIAGLAALGHWDPDQTNTHLGPFGNGHRGDLSRLVVDRDGNTTTSVVAPRLKAS
DLRGRALVVHAGGDTYSDTPPLGGGGARIACGVGS

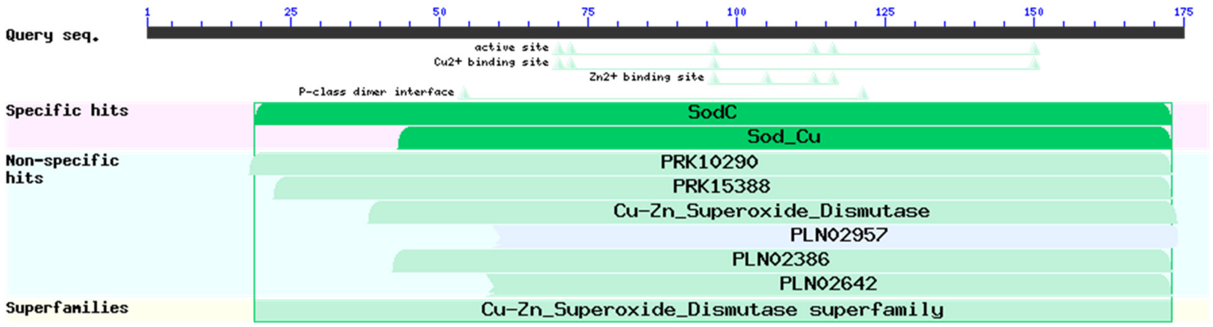

Supplementary Figure S1. Sequence of gene of interest *sodC* obtained from the *S. elongatus* PCC 9311 used in this study (above). *SodC* protein sequence identified from the *Synechococcus elongatus* PCC 9311 has active site and copper-binding residue histidine at positions 70,72, 96,113,116, and 150, and zinc-binding residues histidine and aspartic acid at positions 96, 105, 113, and 116 shows similarity with the other eukaryotic *sodC* at the conserved residues, which suggests that the sequence is strongly associated with Cu/Zn SOD (below).

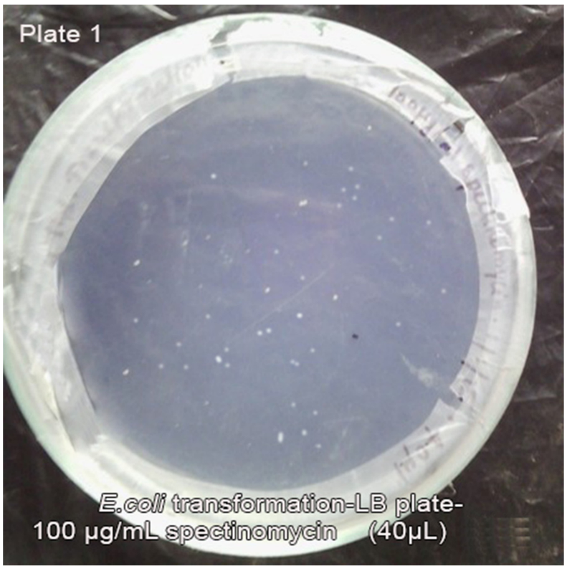

Supplementary Figure S2. Colonies in plate shows the *sodC* transformation in *E. coli* TOP10 in medium containing antibiotic spectinomycin.

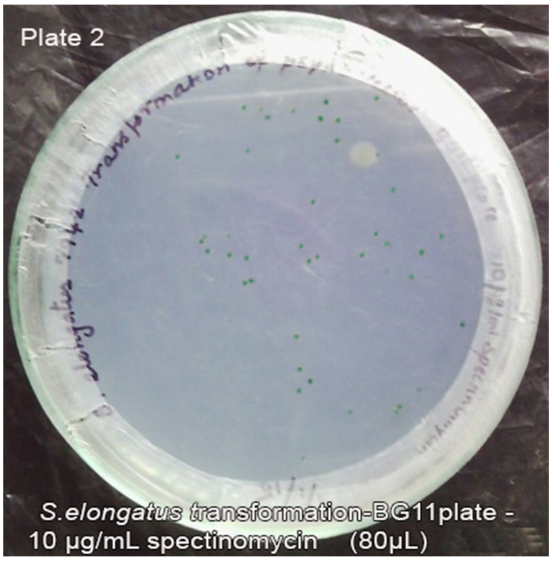

Supplementary Figure S3. Colonies in plate shows the *sodC* transformation in *S. elongatus* PCC 7942 in medium containing antibiotic spectinomycin.

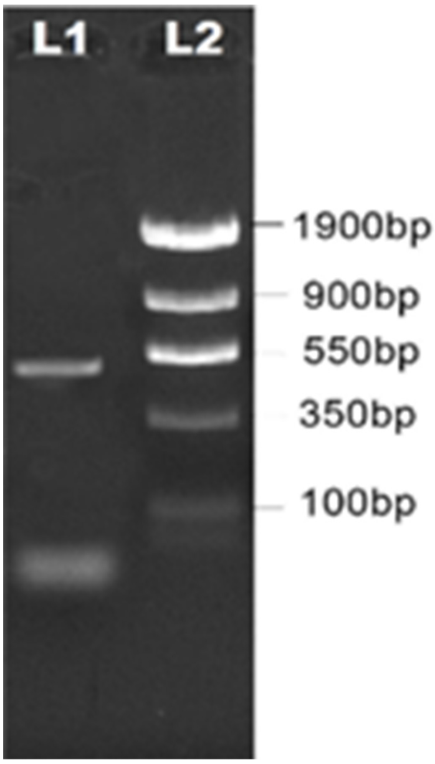

Supplementary Figure S4. PCR confirmation of *sodC* gene integration at neutral sites of *S. elongatus* PCC 7942. Genomic DNA was extracted from transformants grown in a medium containing antibiotic spectinomycin, and PCR was conducted employing specific primers, which anneals at *sodC* region specific for GOI producing a fragment with approximately 520 bp. L1, amplified *sodC* gene from transformants; L2, molecular weights marker.
